# Manual of Pathology

**Published:** 1900-10

**Authors:** 


MANUAL OF PATHOLOGY, Including Bacteriology, the Technic of Post-Mortems, and Methods of Pathologic Research. By W. M. LATE COPLIN, M. D., Professor of Pathology and Bacteriology, Jefferson Medical College, Philadelphia; Pathologist to Jefferson Medical College Hospital and to the Philadelphia (Blockley) Hospital; Bacteriologist to the Pennsylvania State Board of Health. Third edition, revised and enlarged. 330 illustrations and 7 colored plates. Octavo, 846 pages. Philadelphia: P. Blakiston's Son & Co. [Price, $3.50 net.]

From a small beginning, one of the most complete works on pathology has sprung forth and has taken the place in the physician's library of some of the standard European works on this subject. During the winter of 1894 and 1895 the author published in serial form abstracts of his lectures, entitled "Lectures on Pathology" and at the close of the college session these were bound into a volume and placed upon the market. Merit found for them a ready sale and the edition was soon exhausted. This volume contained 250 pages and 51 illustrations. The second edition, published about a year afterward, contained 638 pages and 268 illustrations, and this the third edition, contains 846 pages, and 330 illustrations. This edition has been thoroughly revised, many of the chapters rewritten, new chapters added and a general overhauling has taken place. New illustrations have been added wherever necessary to elucidate the text and some nicely executed colored plates have been introduced.

The work is divided into three parts: Part I. dealing with technic; Part II. with general pathology, and Part III. with special pathology.

Under the chapters on technic—the author describes the methods of making a post-mortem examination, the instruments needed, arrangement of the post-mortem room, the index book, and then gives a very complete guide for the external and internal examination of the body. The different organs are described, and the methods of preserving them for future reference.

Under histologic methods, the author describes the fixing methods in vogue, the infiltration of the tissues, section cutting, staining and then takes up the microscope, describing its component parts and then gives some very useful rules for the use of the microscope. In the same careful, methodical manner is considered the bacteriological technic, the different cultures, inoculation of animals, Widal's test for typhoid fever, the examination of air, water and other fluids.

In chapter IV. the urine is examined, the collecting, centrifuging, examination of the organism and unorganised sediment, and the crystalline sediment are all very carefully explained. The technic of sputum examination occupies Chapter V. and the various epithelium, blood corpuscles and crystals are explained and illustrated.

Part II. deals with general pathology. Chapter I. discusses development, growth, starvation, malformation, disease, pathogenesis; the causes of disease are considered as the thermal, the traumatic and the bacterial. This is an interesting chapter, well written, and shows the author to be a good philosopher, as well as a pathologist.

In Chapter II. the author describes hypertrophy, hyperplasia, metaplasia, atrophy and allied disorders. Then follow chapters on infiltration, desquamation, necrosis, circulatory disturbances, inflammation and repair, the tumors, infective granulomata and temperature changes.

Part III. takes up special pathology. The chapter on the blood is well written, profusely illustrated, and cannot help but give to the student a good, practical understanding of this most important subject. The various blood instruments are described and illustrated and the good points of each are indicated. The chapters following are on the spleen, lymph glands, thymus body, serous membranes, the vascular system, organs of respiration, the liver, pancreas, muscles, bones and nervous system.

These subjects are all handled in the same masterful manner as the preceding chapters, are systematically arranged, carefully and accurately described and well illustrated. The colored plates on the lungs, kidney and bladder are handsome. Many of the subjects are tabulated, making them easily referred to by the reader; for instance, that on the blood, on page 448, is an excellent table. The work throughout shows very careful study and it can be cheerfully commended to student and physician alike.

The index is very complete and sensible—a very important adjunct to a book, inasmuch as it serves as a key for unlocking the contents. In this volume of 51 pages are devoted to the index, which gives some little hint as to the care displayed in the editing of the subject matter. W. C. K.

